# The Formation and Phase Stability of A-Site High-Entropy Perovskite Oxides

**DOI:** 10.3390/ma16062214

**Published:** 2023-03-09

**Authors:** Junzhan Zhang, Shangyi Liu, Zhifeng Tian, Ying Zhang, Zongmo Shi

**Affiliations:** 1 College of Materials Science and Engineering, Xi’an University of Architecture and Technology, Xi’an 710055, China; 2 Shaanxi Key Laboratory of Nano Materials and Technology, Xi’an University of Architecture and Technology, Xi’an 710055, China

**Keywords:** high-entropy perovskite oxides, size disorder, configurational entropy, tolerance factor, electronegativity difference

## Abstract

High entropy perovskite oxides (HEPOs) were a class of advanced ceramic materials, which had attracted much scientific attention in recent years. However, the effect of factors affecting the phase stability of high entropy perovskite oxides was still controversial. Herein, 17 kinds of A-site HEPOs were synthesized by solid-state methods, and several criteria for the formation of HEPOs and phase stability were investigated. Single-phase solid solutions were synthesized in 12 kinds of subsystems. The results show that the phase stability of a single-phase solid solution was affected by the size disorder and configurational entropy. The electronegativity difference was the key parameter to predict the evolution of the cubic/tetragonal phase, rather than the tolerance factor. Cubic HEPOs were easily formed when the electronegativity difference was <0.4, while the tetragonal HEPOs were easily formed when the electronegativity difference was ≥0.4. This study can further broaden the family of HEPOs and is expected to design the phase stability of HEPOs through electronegativity difference.

## 1. Introduction

Since 2004, the research into high-entropy alloys (HEAs) has received great attention due to their vast compositional space and has shown remarkable physical (magnetic and conductivity) and mechanical (strength, hardness and wear resistance) properties [1,2,3]. The HEAs were defined as multi-component alloys which were formed by five or more elements with equal or close to equal atomic fractions [1]. In 2015, Rost et al. were the first to synthesize entropy-stabilized oxides (MgCoNiCuZn)O with a rock salt structure, extending high-entropy materials from alloys to oxides [4]. At present, a large number of high-entropy oxides (HEOs) with different structures were synthesized, including a fluorite [5,6,7,8], pyrochlore [9,10,11,12], spinel [13,14,15,16], and perovskite structure [17,18,19,20,21], and so on [22,23]. Nevertheless, many researchers have committed to exploring new possibilities of entropy-stabilized HEO systems.

Among the typical perovskite oxides, barium titanate (BaTiO_3_) has been widely applied in piezoelectric [24,25], dielectric [26,27,28], and ferroelectric fields [29,30,31], so the high-entropy perovskite oxides (HEPOs) based on BaTiO_3_ have obtained much attention [32,33]. Jiang et al. designed a series of B-site HEPOs with a cubic phase and proposed that the phase stability of a single cubic solid solution was related to the tolerance factor [34]. Chen et al. prepared a variety of B-site HEPOs with a single cubic phase using the strategy of valence combination and proposed that the cation valence difference would affect the phase stability of ordered structures [35,36]. In addition, Sarkar et al. successfully designed high entropy system (Gd_0.2_La_0.2_Nd_0.2_Sm_0.2_Y_0.2_)(Co_0.2_Cr_0.2_Fe_0.2_Mn_0.2_Ni_0.2_)O_3_ and confirmed the role of entropy in the phase stability of a single-phase solid solution [37]. Nevertheless, the phase stability of single-phase HEPOs was not determined by a single factor but was more likely to be in a complex situation. Thus, there was still controversy about the influencing factors on the phase stability of single-phase HEPOs. In addition, the present status of this research is mainly focused on the B-site HEPOs, and most of them are cubic structures without piezoelectricity. Adjusting the phase structure of HEPOs by designing their composition will have great potential and advantages. It is expected to obtain high entropy perovskite ceramics with better ferroelectric and piezoelectric properties.

In the present work, based on BaTiO_3_ (BT), SrTiO_3_ (ST), CaTiO_3_ (CT), PbTiO_3_ (PT), and (Bi_0.5_Na_0.5_)TiO_3_ (BNT), 17 kinds of A-site HEPOs were designed and synthesized by the solid-state method. The phase compositions of the HEPOs were characterized in detail. The scanning electron microscope (SEM) image and energy dispersive spectroscopy (EDS) mapping showed that the element distribution of HEPOs with different molar ratios is different. Moreover, the factors including size disorder, configuration entropy, tolerance factor, and electronegativity difference, affected the formation of a single-phase solid solution, and the phase stability was discussed in detail.

## 2. Experimental Procedure

The following powders BaCO_3_ (99.95%), CaCO_3_ (99.95%), SrCO_3_ (99.95%), PbO (99.95%), MgO (99.95%), Bi_2_O_3_ (99.95%), Y_2_O_3_ (99.95%), Na_2_CO_3_ (99.95%), K_2_CO_3_ (99.95%), and TiO_2_ (99.95%) purchased from Aladdin reagent company were used as starting materials. HEPOs were synthesized by a solid-state method. The powders were weighed according to the stoichiometric compounds shown in Table 1. The obtained powders were mixed homogeneously by ball-milling for 6 h at a rotation speed of 300 r/min using anhydrous ethanol as a medium. After drying, the mixed powders were pressed into discs with a diameter of 20 mm and a thickness of about 10 mm under a uniaxial pressure of 50 MPa. Finally, the discs were calcined at 1000 °C for 3 h in a muffle furnace (KSL-1200X; Kejing Company, Hefei, China) with a heating rate of 3 °C/min and cooled naturally.

The phase composition of the HEPOs was analyzed using X-ray diffraction (XRD, DMAX UItima IV, Rigaku, Japan) with Cu Kα (λ = 1.5418 Å) as the radiation source. Data were digitally recorded in a continuous scan in the range of angle (2*θ*) from 20° to 90°. A scanning electron microscope (SEM, GEMINI SEM 500; Zeiss, Jena, Germany), equipped with an energy dispersive X-ray spectrometer (EDS, UltimMax100, UK), was used to characterize the morphology and element distribution of the HEPOs.

## 3. Results and Discussion

**Figure 1:**
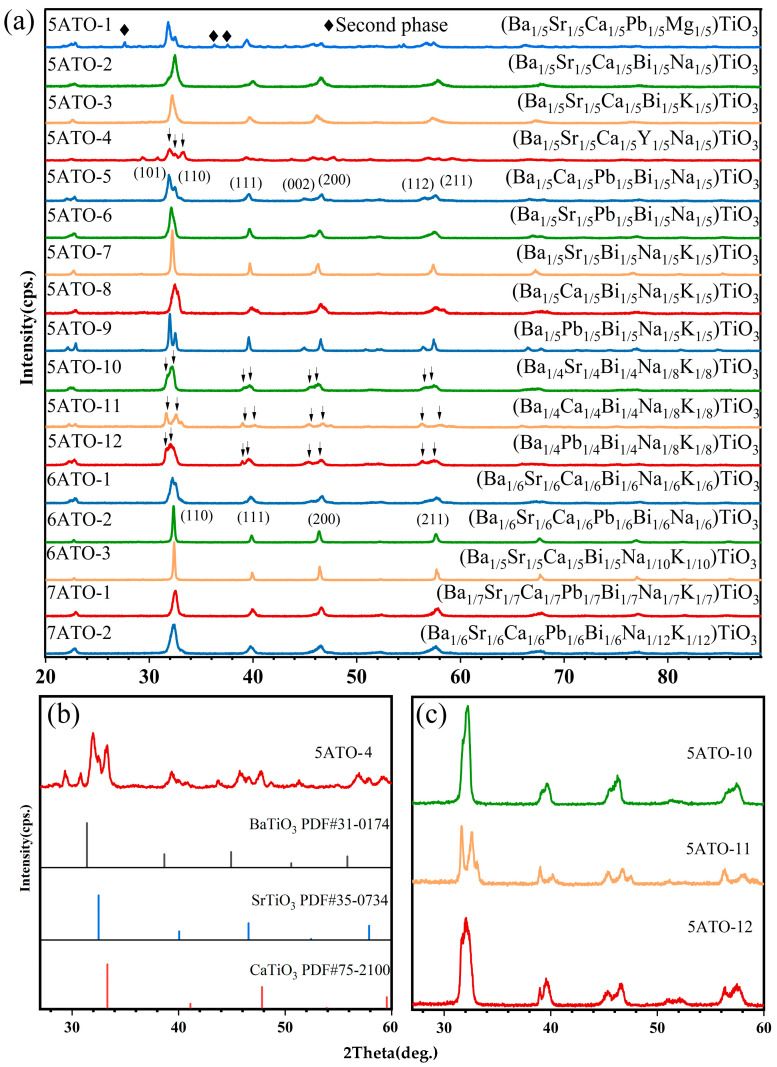
XRD patterns of (**a**) 17 kinds of HEPOs, (**b**) (Ba_1/5_Sr_1/5_Ca_1/5_Y_1/5_Na_1/5_)TiO_3_ (5ATO-4), (**c**) (Ba_1/4_Sr_1/4_Bi_1/4_Na_1/8_K_1/8_)TiO_3_ (5ATO-10), (Ba_1/4_Ca_1/4_Bi_1/4_Na_1/8_K_1/8_)TiO_3_ (5ATO-11), and (Ba_1/4_Pb_1/4_Bi_1/4_Na_1/8_K_1/8_)TiO_3_ (5ATO-12).

Figure 1a shows the XRD patterns of 17 kinds of HEPOs. It is found that three kinds of six-component perovskite oxides (6ATO-1, 6ATO-2, and 6ATO-3) and two kinds of seven-component perovskite oxides (7ATO-1 and 7ATO-2) crystallize as single-phase solid solutions. However, for the five-component perovskite oxides, except 5ATO-1, 5ATO-4, 5ATO-10, 5ATO-11, and 5ATO-12, the others form single-phase solid solutions.

For 5ATO-1, a solid solution with a perovskite structure is basically formed, but there is a small amount of the second phase. As shown in Figure 1b, the peaks in the 2*θ* range of 31–34°, 39–42°, 46–49°, and 57–60° are composed of three peaks, respectively, which correspond to the standard card of BaTiO_3_ (PDF#31-0174), SrTiO_3_ (PDF#35-0734), and CaTiO_3_ (PDF#75-2100), indicating that three-phase (BT, ST, and CT) coexist in 5ATO-4. In addition, a small number of impurities are present in 5ATO-4.

As shown in Figure 1c, by comparing the peaks in the 2*θ* range of 31–33°, 38–40°, 45–47°, and 56–58° of 5ATO-10, 5ATO-11, and 5ATO-12 with the standard card of BaTiO_3_ (PDF#31-0174), SrTiO_3_ (PDF#35-0734), CaTiO_3_ (PDF#75-2100), and PbTiO_3_ (PDF#40-0099), the occurrences of two diffraction peaks during each 2*θ* range prove that a single-phase solid solution cannot be formed. At the 2*θ* range of 31–33°, the separation of the 5ATO-11 peak is farther than that of 5ATO-10 and 5ATO-12. This is because the radius of Ca^2+^ (1.34 Å) is less than Sr^2+^ (1.44 Å) and Pb^2+^ (1.49 Å).

**Figure 2:**
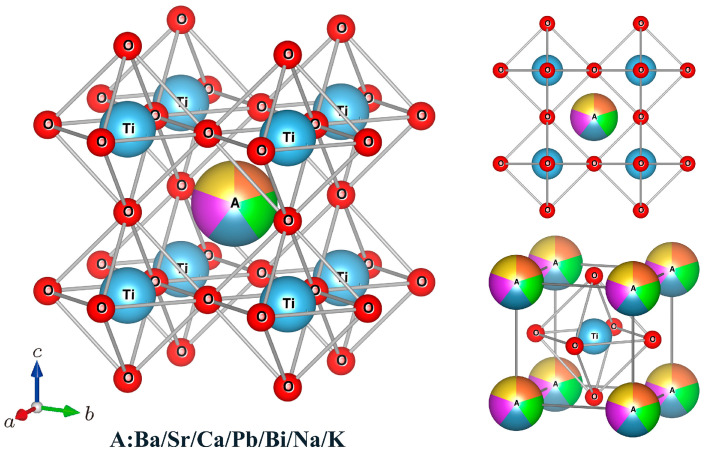
Crystal structure of HEPOs with multiple elements at A-site. The red sphere represents oxygen ion, the blue sphere represents titanium cation at B-site, and the colored ball represents the multi-component cations at A-site.

For perovskite oxides ABO_3_, the A-site ions occupy the corners and the B-site ions occupy the body center of the cube, while the oxygen ions locate the face centers. B-site ions have 6-fold coordination and form BO_6_ octahedrons with oxygen ions, while the A ions have 12-fold coordination [38]. For A-site HEPOs, multiple cations occupy the A-site randomly, but the crystal structure remains unchanged and stable. Figure 2 displays the crystal structure of HEPOs with multiple elements at A-site. According to the classical Hume-Rothery (H-R) rules, the cation size mismatch plays an important role in the formation of a complete solid solution [39]. To form a complete solid solution, the cation radii should have little difference. When the cation size mismatch exceeds 15%, the solid solution becomes unstable [40]. Therefore, to form A-site HEPOs with stable crystal structures, the A-site cation radii should show little difference.

Zhang et al. proposed that size disorder characterized the solid solution behavior of the constituent elements in the multi-component alloys [41]. Size disorder was also utilized to judge the formation of B-site HEPOs by Jiang et al. [34]. Thus, size disorder ($\delta_{A}^{r}$) is used to characterize the cation size difference of A-site HEPOs in our work, and it can be defined as follows:

(1) $$\delta_{A}^{r}=\sqrt{\sum_{i=1}^{N}x_{i}{\left[1-\frac{R_{i}}{\sum_{i=1}^{N}x_{i}R_{i}}\right]}^{2}}$$

where $x_{i}$ and $R_{i}$ are the mole fraction and the radius of the *i* cation at A-site, respectively. The size disorder of 17 kinds of HEPOs is shown in Table 1. It can be seen that the $\delta_{A}^{r}$ values ranged from 5.73% to 8.59% except for 5ATO-1 and 5ATO-4. Single-phase HEPOs are not formed in two compositions with high $\delta_{A}^{r}$ (18.30% for 5ATO-1 and 12.67% for 5ATO-4, respectively). Miracle et al. indicated that the $\delta_{A}^{r}$ values have an upper limit of 6.5% for the formation of single-phase HEAs [42]. For 5ATO-3 and 5ATO-8, the $\delta_{A}^{r}$ values even exceed 8%. Thus, it seems that this upper limit should be higher for A-site HEPOs. The results are consistent with some synthesized HEPOs [17,43].

Furthermore, it is noted that the $\delta_{A}^{r}$ value of 5ATO-10, 5ATO-11, and 5ATO-12 is 7.15%, 8.52%, and 6.87%, respectively (albeit smaller than 7.43% of 5ATO-7, 8.59% of 5ATO-8, and 7.19% of 5ATO-9, respectively). However, 5ATO-10, 5ATO-11, and 5ATO-12 cannot form a single-phase solid solution. Thus, the cation size difference is probably a necessary criterion, but not a sufficient criterion for HEPOs to obtain a single-phase solid solution.

The multiple elements at the A-site (Ba, Sr, Bi, Na, and K) of 5ATO-10 and 5ATO-7 are the same, but the mole fractions of the five elements are different. Therefore, the configuration entropy of 5ATO-10 and 5ATO-7 are different. Further consistent with this situation are 5ATO-8 and 5ATO-11, 5ATO-9 and 5ATO-12. The configuration entropy ($\Delta S_{mix}$) is defined as follows [44]:

(2) $$\Delta S_{mix}=-R\sum_{i=1}^{N}x_{i}lnx_{i}$$

where *R* is the gas constant; $x_{i}$ is the molar fraction of the *i* cation at A-site; and *N* represents the number of components at A-site. The calculated configuration entropy by the corresponding stoichiometric formula is given in Table 1. The $\Delta S_{mix}$ value of 5ATO-7, 5ATO-8, and 5ATO-9 is 1.609R, while that of 5ATO-10, 5ATO-11, and 5ATO-12 is 1.560R. Rost et al. found that the formation of a high-entropy solid solution was affected by the molar fraction of cations, and indicated that the configuration entropy was an important driving force for the formation of a single phase [3]. According to the concept of high entropy, the $\Delta S_{mix}$ value should be higher than 1.609R [45]. Therefore, the low $\Delta S_{mix}$ value is a factor that affects 5ATO-10, 5ATO-11, and 5ATO-12 and cannot form a single-phase solid solution.

**Figure 3:**
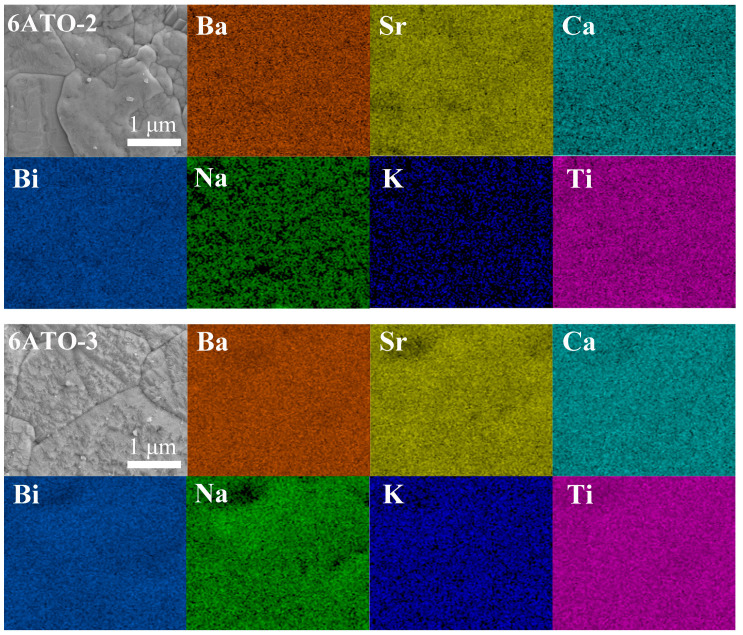
SEM images and corresponding EDS sample mapping of 6ATO-2 and 6ATO-3.

The multiple elements at A-site (Ba, Sr, Ca, Bi, Na, and K) of 6ATO-2 and 6ATO-3 are the same, but the mole fractions of the six elements are different. For these two kinds of HEPOs, a single-phase solid solution is formed, and their configuration entropy is greater than 1.7R. Figure 3 shows SEM images and corresponding EDS sample mapping of 6ATO-2 and 6ATO-3. It can be seen that the elements of Ba, Sr, Ca, Bi, Na, K, and Ti are randomly distributed in the samples of 6ATO-2 and 6ATO-3. However, the content of Na and K elements is significantly low for sample 6ATO-2. It may be caused by the fact that the sample of 6ATO-2 does not conform to the valence balance [46].

To further investigate the phase structure, 12 kinds of single-phase HEPOs were analyzed in detail. It is worth noting that there are different phases among these single-phase solid solutions: cubic perovskite structure and tetragonal perovskite structure.

**Figure 4:**
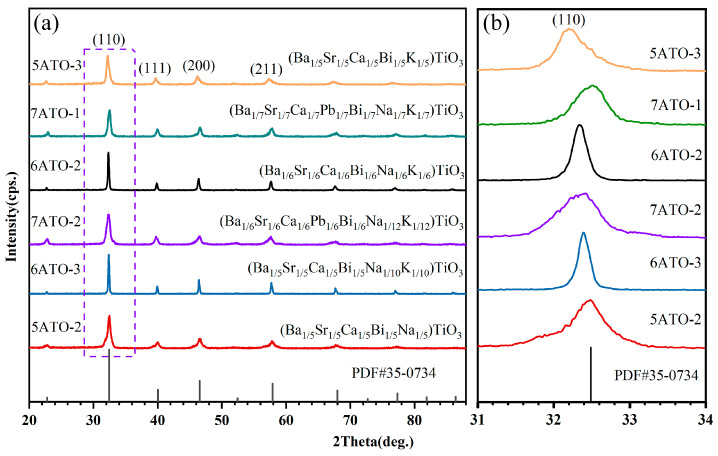
(**a**) XRD patterns of cubic perovskite oxides, (**b**) Zoom in view of the XRD patterns between 31° and 34°.

Figure 4a shows the XRD pattern of the six kinds of cubic HEPOs. It can be seen that the six oxides exhibit typical diffraction peaks of (110), (111), (200), and (211), which correspond to the standard card of SrTiO_3_ (PDF#35-0734) [47], proving that they possess cubic perovskite structure.

In addition, as shown in Figure 4b, XRD patterns in the 2θ range from 31° to 34° were enlarged. It is worth noting that the diffraction peaks shift to the left with the increase in the average radius of A-site cations, with only one exception (7ATO-1). The average radius of A-site ions of 5ATO-2, 5ATO-3, 6ATO-2, 6ATO-3, 7ATO-1, and 7ATO-2 are 1.432 Å, 1.457 Å, 1.463 Å, 1.467 Å, 1.470 Å, and 1.482 Å, respectively. The diffraction peaks shift to a lower angle, indicating the increase in lattice value. The lattice constants of 12 kinds of single-phase HEPOs are summarized in Table S1 (Supplementary Materials). When the elements with larger ion radii enter the crystal lattice, they will cause the expansion of the cell. This is consistent with Bragg’s law [48]. For 7ATO-1, a slight deviation from the valence balance may cause lattice distortion and lead to exceptional uncertainty [46].

**Figure 5:**
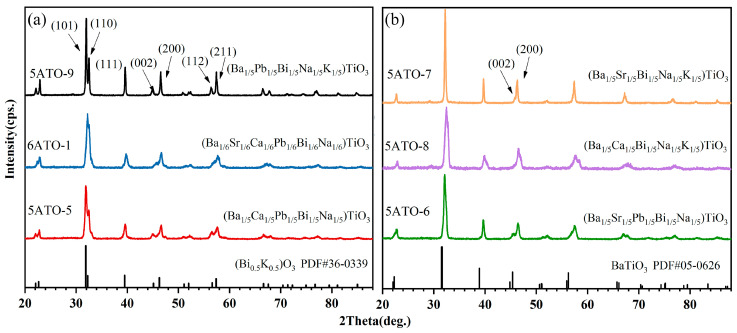
XRD patterns of the six samples which possess tetragonal perovskite structure: (**a**) 5ATO-5, 5ATO-9, and 6ATO-1, (**b**) 5ATO-6, 5ATO-7, and 5ATO-8.

As shown in Figure 5a, for the three oxides (5ATO-5, 5ATO-9, and 6ATO-1), the diffraction peaks at 2*θ* = 31°, 32°, 40°, 45°, 46°, 56°, and 57° correspond to (101), (110), (111), (002), (200), (112), and (211) planes, indicating that the three oxides correspond to the standard card of tetragonal (Bi_0.5_K_0.5_)TiO_3_ (PDF#36-0339) [49]. The XRD patterns of the other three oxides (5ATO-6, 5ATO-7, and 6ATO-8) are given in Figure 5b. The diffraction peak at 2*θ* = 32°, as well the split of the diffraction peak at 2*θ* = 44.75° into two peaks of (002) and (200), indicate that the three oxides correspond to the standard card of tetragonal BaTiO_3_ (PDF#05-0626) [50].

According to the lattice constants summarized in Table S1, the values of *c*/*a* of six oxides with tetragonal structure are calculated. The values of *c*/*a* are 1.035, 1.021, and 1.033 for 5ATO-5, 6ATO-1, and 5ATO-9, respectively, which are close to the value of *c*/*a* of (Bi_0.5_K_0.5_)TiO_3_ (1.024). The values of *c*/*a* are 1.016, 1.008, and 1.008 for 5ATO-6, 5ATO-7, and 5ATO-8, respectively, which are close to the value of *c*/*a* of BaTiO_3_ (1.011). This is consistent with the corresponding result of XRD results.

For perovskite oxides, Goldschmidt proposed a tolerance factor (*t*) to characterize the stability and cubic symmetry deviation of perovskite structure [51]. The value is defined using the following formula:

(3) $$t=\frac{R_{A}+R_{O}}{\sqrt{2}\left(R_{B}+R_{O}\right)}$$

where $R_{A}$ and $R_{B}$ are the ionic radii of the cations at A and B sites, respectively, and $R_{O}$ is the radius of the oxygen ion. A cubic phase is likely to be stable if 0.9 ≤ *t* ≤ 1.0, while a tetragonal phase may be formed if *t* > 1.0 [30]. In the case of multiple cations at the A-site, the average ionic radius is considered. The calculated *t* values of the samples are given in Table 1. We find that the *t* values of each A-site HEPO are in the range of 0.98–1.02. In fact, for six oxides possessing a cubic perovskite structure (5ATO-2, 5ATO-3, 6ATO-1, 6ATO-2, 7ATO-1, and 7ATO-2), the values are 1.00, 1.02, 0.99, 1.00, 1.00, and 0.98, respectively; meanwhile, for six oxides possessing tetragonal perovskite structure (5ATO-5, 5ATO-6, 5ATO-7, 5ATO-8, 5ATO-9, and 6ATO-1), the values are 0.99, 1.00, 1.02, 1.00, 1.01, and 0.99, respectively. Thus, there is no correlation between the tolerance factor and the phase structure, i.e., the *t* value cannot be used to predict whether the HEPOs form a cubic perovskite phase or a tetragonal perovskite phase.

For HEAs, there is an obvious correlation between the electronegativity difference and whether the HEAs form body-centered cubic (BCC) or face-centered cubic (FCC) structures [52]. Herein, we introduce the concept of electronegativity difference into HEPOs. For A-site HEPOs, the electronegativity difference ($\delta_{A}^{e}$) is defined as follows:

(4) $$\delta_{A}^{e}=\sqrt{\sum_{i=1}^{N}x_{i}{\left[1-\frac{e_{i}}{\sum_{i=1}^{N}x_{i}e_{i}}\right]}^{2}}$$

where $e_{i}$ and $x_{i}$ are the electronegativity and the mole fraction of the *i* cation at A-site, respectively. According to the Pauling electronegativity of each element, the calculated $\delta_{A}^{e}$ values are listed in Table 1. It can be found that, except for 7ATO-1 and 7ATO-2, the $\delta_{A}^{e}$ values of other cubic perovskite oxides (5ATO-2, 5ATO-3, 6ATO-2, 6ATO-3) are less than 0.4 (0.37, 0.39, 0.36, and 0.38, respectively). The $\delta_{A}^{e}$ values of all tetragonal perovskite oxides (5ATO-5, 5ATO-6, 5ATO-7, 5ATO-8, 5ATO-9, and 6ATO-1) are higher than 0.4 (0.43, 0.44, 0.40, 0.40, 0.46, and 0.44, respectively). Pb is higher than other meter elements selected in this study. Therefore, the $\delta_{A}^{e}$ values of the four oxides containing Pb (5ATO-5, 5ATO-6, 5ATO-9, 6ATO-1) are relatively higher, and HEPOs containing Pb prefer to form tetragonal perovskite structures. It is worth noting that the $\delta_{A}^{e}$ values of 5ATO-7 and 5ATO-8 are both 0.40, which is the boundary value. Furthermore, it can be found that the *c*/*a* values of 5ATO-7 and 5ATO-8 are close to 1, indicating that they have a tendency from the tetragonal perovskite phase to the cubic perovskite phase. However, the $\delta_{A}^{e}$ values of 7ATO-1 and 7ATO-2 are 0.44 and 0.45, respectively, because of their Pb-containing, but they form a cubic perovskite phase. The fact that it may have originated from more principal elements at A-site and higher configurational entropy should be investigated in detail later. The prediction for the phase evolution between cubic and tetragonal by the $\delta_{A}^{e}$ values are consistent with the result of this work. It is well known that the degree of the tetragonal phase is related to ferroelectric properties. The high entropy perovskite ceramics prepared by this method are expected to have better ferroelectric properties.

## 4. Conclusions

A series of A-site HEPOs with five components, six components, and seven elements were successfully synthesized by the solid-state method. Subsequently, the values of size disorder, configurational entropy, tolerance factor, and electronegativity difference were calculated to clarify the correlation with the formation and phase stability. The formation of single-phase A-site HEPOs can be predicted by $\delta_{A}^{r}$ and $\Delta S_{mix}$. Oxides 5ATO-1 and 5ATO-4 with higher $\delta_{A}^{r}$ values do not easily form single-phase solid solutions. Meanwhile, 5ATO-10 with low $\Delta S_{mix}$ value cannot form a single-phase solid solution. The *t* values of 12 kinds of A-site HEPOs were in the range of 0.98–1.02, which cannot be used to predict whether the HEPOs form a cubic perovskite phase or a tetragonal perovskite phase. Finally, the $\delta_{A}^{e}$ values were successfully used to predict the phase stability of HEPOs. Cubic HEPOs were easily formed when the electronegativity difference was < 0.4, while the tetragonal HEPOs were easily formed when the electronegativity difference was ≥ 0.4.

## Figures and Tables

**Table 1:** Summary of important results of 17 kinds of HEPOs.

No.	Composition	Phase	$\Delta S_{mix}$	${\bar{R}}_{A}(Å)$	*t*	$\delta_{A}^{r}$	$\delta_{A}^{e}\;$
5ATO-1	(Ba_1/5_Sr_1/5_Ca_1/5_Pb_1/5_Mg_1/5_)TiO_3_	F	1.609R	1.376	0.98	18.30	-
5ATO-2	(Ba_1/5_Sr_1/5_Ca_1/5_Bi_1/5_Na_1/5_)TiO_3_	C		1.432	1.00	6.60	0.37
5ATO-3	(Ba_1/5_Sr_1/5_Ca_1/5_Bi_1/5_K_1/5_)TiO_3_	C		1.482	1.02	8.19	0.39
5ATO-4	(Ba_1/5_Sr_1/5_Ca_1/5_Y_1/5_Na_1/5_)TiO_3_	F		1.372	0.98	12.67	-
5ATO-5	(Ba_1/5_Ca_1/5_Pb_1/5_Bi_1/5_Na_1/5_)TiO_3_	T		1.442	0.99	6.76	0.43
5ATO-6	(Ba_1/5_Sr_1/5_Pb_1/5_Bi_1/5_Na_1/5_)TiO_3_	T		1.462	1.00	5.73	0.44
5ATO-7	(Ba_1/5_Sr_1/5_Bi_1/5_Na_1/5_K_1/5_)TiO_3_	T		1.492	1.02	7.43	0.40
5ATO-8	(Ba_1/5_Ca_1/5_Bi_1/5_Na_1/5_K_1/5_)TiO_3_	T		1.472	1.00	8.59	0.40
5ATO-9	(Ba_1/5_Pb_1/5_Bi_1/5_Na_1/5_K_1/5_)TiO_3_	T		1.502	1.01	7.19	0.46
5ATO-10	(Ba_1/4_Sr_1/4_Bi_1/4_Na_1/8_K_1/8_)TiO_3_	F	1.560R	1.486	1.00	7.15	-
5ATO-11	(Ba_1/4_Ca_1/4_Bi_1/4_Na_1/8_K_1/8_)TiO_3_	F		1.461	1.01	8.52	-
5ATO-12	(Ba_1/4_Pb_1/4_Bi_1/4_Na_1/8_K_1/8_)TiO_3_	F		1.499	1.03	6.87	-
6ATO-1	(Ba_1/6_Sr_1/6_Ca_1/6_Pb_1/6_Bi_1/6_Na_1/6_)TiO_3_	T	1.790R	1.442	0.99	6.17	0.44
6ATO-2	(Ba_1/6_Sr_1/6_Ca_1/6_Bi_1/6_Na_1/6_K_1/6_)TiO_3_	C		1.467	1.00	7.91	0.36
6ATO-3	(Ba_1/5_Sr_1/5_Ca_1/5_Bi_1/5_Na_1/10_K_1/10_)TiO_3_	C	1.748R	1.457	0.99	7.66	0.38
7ATO-1	(Ba_1/7_Sr_1/7_Ca_1/7_Pb_1/7_Bi_1/7_Na_1/7_K_1/7_)TiO_3_	C	1.946R	1.470	1.00	7.33	0.45
7ATO-2	(Ba_1/6_Sr_1/6_Ca_1/6_Pb_1/6_Bi_1/6_Na_1/12_K_1/12_)TiO_3_	C	1.907R	1.463	0.98	7.02	0.44

The sample that cannot form a single phase is labeled as “F”, the sample that can form cubic perovskite is labeled as “C”, and the sample that can form tetragonal perovskite is labeled as “T”. ${\bar{R}}_{A}$ is the average radius of A-site cations, and other parameters are described in detail later.

## Data Availability

The data presented in this study are available on request from the corresponding author.

## Supplementary Materials

The following supporting information can be downloaded at: https://www.mdpi.com/article/10.3390/ma16062214/s1, Table S1: Lattice constants of 12 kinds of single-phase HEPOs.

## References

[1] Yeh J.W., Chen S.K., Lin S.J. (2004). Nanostructured high-entropy alloys with multiple principal elements: Novel alloy design concepts and outcomes. Adv. Eng. Mater..

[2] Cantor B., Chang I., Knight P., Vincent A. (2004). Microstructural development in equiatomic multicomponent alloys. Mater. Sci. Eng. A..

[3] Pavel A.L., Alexander D.F., Samat K.M., Olga S.M. (2023). Manufacturing of metal-diamond composites with high-strength CoCrCu_x_FeNi high-entropy alloy used as a binder. Materials.

[4] Rost C.M., Sachet E., Borman T., Moballegh A., Dickey E.C., Hou D. (2015). Entropy-stabilized oxides. Nat. Commun..

[5] Xue Y., Zhao X., An Y. (2022). High-entropy (La_0.2_Nd_0.2_Sm_0.2_Eu_0.2_Gd_0.2_)_2_Ce_2_O_7_: A potential thermal barrier material with improved thermo-physical properties. J. Adv. Ceram..

[6] Xie H.H., Li J.S., Yang S.Z. (2021). Microstructures and dielectric properties of novel (La_0.2_Pr_0.2_Nd_0.2_Sm_0.2_Eu_0.2_)_2_Ce_2_O_7_ high entropy ceramics. J. Mater. Sci. Mater. Electron..

[7] Andrew J., Wright A., Wang Q.Y. (2020). From high-entropy ceramics to compositionally-complex ceramics: A case study of fluorite oxides. J. Eur. Ceram. Soc..

[8] Spiridigliozzi L., Ferone C., Cioffi R. (2021). A simple and effective predictor to design novel fluorite-structured high entropy oxides (HEOs). Acta Mater..

[9] Zhu J., Wei M., Xu J. (2022). Influence of order-disorder transition on the mechanical and thermophysical properties of A_2_B_2_O_7_ high-entropy ceramics. J. Adv. Ceram..

[10] Chen Y., Qi J., Zhang M. (2022). Pyrochlore-based high-entropy ceramics for capacitive energy storage. J. Adv. Ceram..

[11] Jia H., Li C., Chen G. (2022). Design and synthesis of high-entropy pyrochlore ceramics based on valence combination. J. Eur. Ceram. Soc..

[12] Wright A.J., Wang Q., Ko S.T. (2020). Size disorder as a descriptor for predicting reduced thermal conductivity in medium- and high-entropy pyrochlore oxides. Scr. Mater..

[13] Xu Y., Xu X., Bi L. (2022). A high-entropy spinel ceramic oxide as the cathode for proton-conducting solid oxide fuel cells. Adv. Ceram..

[14] Ma J., Zhao B., Xiang H. (2022). High-entropy spinel ferrites MFe_2_O_4_ (M = Mg, Mn, Fe, Co, Ni, Cu, Zn) with tunable electromagnetic properties and strong microwave absorption. J. Adv. Ceram..

[15] Huang C.Y., Huang C.W., Wu M.C. (2021). Atomic-scale investigation of lithiation/delithiation mechanism in high-entropy spinel oxide with superior electrochemical performance. Chem. Eng. J..

[16] Duan C.Q., Tian K., Li X. (2021). New spinel high-entropy oxides (FeCoNiCrMnXLi)_3_O_4_ (X = Cu, Mg, Zn) as the anode material for lithium-ion batteries. Ceram. Int..

[17] Zheng Y., Zou M., Zhang W. (2021). Electrical and thermal transport behaviours of high-entropy perovskite thermoelectric oxides. J. Adv. Ceram..

[18] Zhou S.Y., Pu Y.P., Zhao X.Y. (2022). Dielectric temperature stability and energy storage performance of NBT-based ceramics by introducing high-entropy oxide. J. Am. Ceram. Soc..

[19] Liu Z., Xu S., Li T. (2021). Microstructure and ferroelectric properties of high-entropy perovskite oxides with A-site disorder. Ceram. Int..

[20] Wang Q., Zhang Q., Wang G. (2022). High-entropy La(Fe_0.2_Co_0.2_Ni_0.2_Cr_0.2_Mn_0.2_)O_3_ ceramic exhibiting high emissivity and low thermal conductivity. Int. J. Appl. Ceram. Technol..

[21] Eiselt L., Kruk R., Hahn H. (2023). Hole-doped high entropy ferrites: Structure and charge compensation mechanisms in (Gd_0.2_La_0.2_Nd_0.2_Sm_0.2_Y_0.2_)1−xCaxFeO3. Int. J. Appl. Ceram. Technol..

[22] Su J., Cao Z., Jiang Z. (2022). High entropy oxide nanofiber by electrospun method and its application for lithium battery anode material. Int. J. Appl. Ceram. Technol..

[23] Zhang G., Wu Y. (2022). High-entropy transparent ceramics: Review of potential candidates and recently studied cases. Int. J. Appl. Ceram. Technol..

[24] Zheng T.Y., Han Z., Huang Y.Q. (2021). Piezoelectric calcium/manganese-doped barium titanate nanofibers with improved osteogenic activity. Ceram. Int..

[25] Mahesh M., Bhanuprasad V.V., James A.R. (2013). Enhanced piezoelectric properties and tunability of lead-free ceramics prepared by high-energy ball milling. J. Electron. Mater..

[26] He X., Han F., Liu M. (2020). High-temperature dielectric and relaxation behavior of tantalum-doped sodium bismuth titanate-barium titanate ceramics. J. Electron. Mater..

[27] Zhou J., Li P., Zhang X. (2022). Effect of configurational entropy on dielectric properties of high-entropy perovskite oxides (Ce_0.5_,K_0.5_)_x_[(Bi_0.5_,Na_0.5_)_0.25_Ba_0.25_Sr_0.25_Ca_0.25_]_1-x_TiO_3_. J. Mater. Sci. Mater. Electron..

[28] Li S., Li J., Zhou C. (2022). Research on the dielectric energy storage characteristics of the [(Bi_0.5_Na_0.5_)_0.2_Ba_0.2_Sr_0.2_Ca_0.2_Mg_0.2_]TiO_3_ equal ratio high-entropy ceramics. J. Mater. Sci. Mater. Electron..

[29] Kumar S., Shrivastava V., Thakur O.P. (2023). Cumulative effect of yttrium and tin co-doping on the structural and ferroelectric properties of sol-gel derived barium titanate. J. Sol-Gel. Sci. Technol..

[30] Chawla A., Verma S., Godara S. (2020). Understanding phase segregation using rietveld analysis and the dielectric, ferroelectric properties of Ba_(1-x)_Ca_x_TiO_3_ solid solutions. J. Electron. Mater..

[31] Shang Y., Pu Y., Zhang Q. (2022). Effect of configuration entropy on dielectric relaxor, ferroelectric properties of high-entropy (NaBiBa)_x_(SrCa)_(1-3x)/2_TiO_3_ ceramics. J. Mater. Sci. Mater. Electron..

[32] Zhou S., Pu Y., Zhang Q. (2020). Microstructure and dielectric properties of high entropy Ba(Zr_0.2_Ti_0.2_Sn_0.2_Hf_0.2_Me_0.2_)O_3_ perovskite oxides. Ceram. Int..

[33] Du Q., Yan J., Zhang X. (2020). Phase evolution and dielectric properties of Ba(Ti_1/6_Sn_1/6_Zr_1/6_Hf_1/6_Nb_1/6_Ga_1/6_)O_3_ high-entropy perovskite ceramics. J. Mater. Sci. Mater. Electron..

[34] Jiang S., Hu T., Gild J. (2018). A new class of high-entropy perovskite oxides. Scr. Mater..

[35] Tang L., Li Z.M., Chen K.P. (2020). High-entropy oxides based on valence combinations: Design and practice. J. Am. Ceram. Soc..

[36] Ma J., Chen K.P., Li C. (2021). High-entropy stoichiometric perovskite oxides based on valence combinations. Ceram. Int..

[37] Sarkar A., Djenadic R., Wang D. (2017). Rare earth and transition metal based entropy stabilised perovskite type oxides. J. Eur. Ceram. Soc..

[38] Ramadass N. (1978). ABO_3_-type oxides-their structure and properties-a bird’s eye view. Mater. Sci. Eng..

[39] Wang Z.J., Qiu W.F., Yang Y. (2015). Atomic-size and lattice-distortion effects in newly developed high-entropy alloys with multiple principal elements. Intermetallics.

[40] Eshelby J.D. (1956). The continuum theory of lattice defects. Solid State Phys..

[41] Zhang Y., Zhou Y.J., Lin J.P. (2008). Solid-solution phase formation rules for multi-component alloys. Adv. Eng. Mater..

[42] Miracle D.B., Senkov O.N. (2017). A critical review of high entropy alloys and related concepts. Acta. Mater..

[43] Teng Z., Tan Y., Zeng S. (2021). Preparation and phase evolution of high-entropy oxides A_2_B_2_O_7_ with multiple elements at A and B sites. J. Eur. Ceram. Soc..

[44] Sarkar A., Breitung B., Hahn H. (2020). High entropy oxides: The role of entropy, enthalpy and synergy. Scr. Mater..

[45] Akrami S., Edalati P., Fuji M. (2021). High-entropy ceramics: Review of principles, production and applications. Mater. Sci. Eng. R Rep..

[46] Liu J., Ren K., Ma C. (2020). Dielectric and energy storage properties of flash-sintered high-entropy (Bi_0.2_Na_0.2_K_0.2_Ba_0.2_Ca_0.2_)TiO_3_ ceramic. Ceram. Int..

[47] Pu Y.P., Zhang Q.W., Li R. (2019). Dielectric properties and electrocaloric effect of high-entropy (Na_0.2_Bi_0.2_Ba_0.2_Sr_0.2_Ca_0.2_)TiO_3_ ceramic. Appl. Phys. Lett..

[48] Bragg W. (1913). The reflection of X-rays by crystals. Nature.

[49] Krad I., Bidault O., Geoffroy N. (2016). Preparation and characterization of K_0.5_Bi_0.5_TiO_3_ particles synthesized by a stirring hydrothermal method. Ceram. Int..

[50] Li X., Ma J.X., Chen K.P. (2022). Design and investigate the electrical properties of Pb(Mg_0.2_Zn_0.2_Nb_0.2_Ta_0.2_W_0.2_)O_3_-PbTiO_3_ high-entropy ferroelectric ceramics-sciencedirect. Ceram. Int..

[51] Goldschmidt V.M. (1926). Die gesetze der krystallochemie. Naturwissenschaften.

[52] Nong Z.S., Zhu J.C., Cao Y. (2014). Stability and structure prediction of cubic phase in as cast high entropy alloys. Mater. Sci. Technol..

