# Supplementary material for: The Formation and Phase Stability of A-Site High-Entropy Perovskite Oxides
Source: Materials (Basel). 2023 Mar 9;16(6):2214. doi: 10.3390/ma16062214 (PMC10052205; doi:10.3390/ma16062214)
Supplement: Supplementary file 1 [file materials-16-02214-s001.zip › materials-2240745-supplementary.pdf]

Table S1. Lattice constants of 12 kinds of single-phase HEPOs.

No.	Composition	*a*(Å)	*b*(Å)	*c*(Å)
5ATO-2	$(Ba_{1/5}Sr_{1/5}Ca_{1/5}Bi_{1/5}Na_{1/5})TiO_3$	3.902	3.902	3.902
5ATO-3	$(Ba_{1/5}Sr_{1/5}Ca_{1/5}Bi_{1/5}K_{1/5})TiO_3$	3.932	3.932	3.932
5ATO-5	$(Ba_{1/5}Ca_{1/5}Pb_{1/5}Bi_{1/5}Na_{1/5})TiO_3$	3.894	3.894	4.030
5ATO-6	$(Ba_{1/5}Sr_{1/5}Pb_{1/5}Bi_{1/5}Na_{1/5})TiO_3$	3.911	3.911	3.975
5ATO-7	$(Ba_{1/5}Sr_{1/5}Bi_{1/5}Na_{1/5}K_{1/5})TiO_3$	3.922	3.922	3.952
5ATO-8	$(Ba_{1/5}Ca_{1/5}Bi_{1/5}Na_{1/5}K_{1/5})TiO_3$	3.909	3.909	4.039
5ATO-9	$(Ba_{1/5}Pb_{1/5}Bi_{1/5}Na_{1/5}K_{1/5})TiO_3$	3.901	3.901	3.932
6ATO-1	$(Ba_{1/6}Sr_{1/6}Ca_{1/6}Pb_{1/6}Bi_{1/6}Na_{1/6})TiO_3$	3.889	3.889	3.969
6ATO-2	$(Ba_{1/6}Sr_{1/6}Ca_{1/6}Bi_{1/6}Na_{1/6}K_{1/6})TiO_3$	3.913	3.913	3.913
6ATO-3	$(Ba_{1/5}Sr_{1/5}Ca_{1/5}Bi_{1/5}Na_{1/10}K_{1/10})TiO_3$	3.907	3.907	3.907
7ATO-1	$(Ba_{1/7}Sr_{1/7}Ca_{1/7}Pb_{1/7}Bi_{1/7}Na_{1/7}K_{1/7})TiO_3$	3.905	3.905	3.905
7ATO-2	$(Ba_{1/6}Sr_{1/6}Ca_{1/6}Pb_{1/6}Bi_{1/6}Na_{1/12}K_{1/12})TiO_3$	3.910	3.910	3.910
